# Functional dentition is a modifier of the association between vitamin D and the frailty index among Chinese older adults: a population-based longitudinal study

**DOI:** 10.1186/s12877-022-02857-3

**Published:** 2022-02-28

**Authors:** Miao Dai, Jirong Yue, Jingyi Zhang, Hui Wang, Chenkai Wu

**Affiliations:** 1 Department of Geriatrics and National Clinical Research Center for Geriatrics, West China Hospital of Sichuan University, Chengdu, 610041 Sichuan China; 2 Jiujiang First People’s Hospital, Jiujiang, 332000 Jiangxi China; 3 Dartmouth College, Hanover, NH 03755 USA; 4 Global Health Research Center, Duke Kunshan University, Kunshan, 215300 Jiangsu China

**Keywords:** Vitamin D, Dentition, Frailty, Old adult, Cohort study

## Abstract

**Backgrounds:**

Although vitamin D and dentition status are each associated with frailty, their combined effects on frailty have not been studied. This study aimed to evaluate the combined effects of vitamin D and dentition status on frailty in old Chinese adults.

**Methods:**

Baseline data were obtained from the 2011–2012 wave of the Chinese Longitudinal Healthy Longevity Survey. A total of 1074 participants ≥65 years who were non-frail or prefrail at baseline were included; follow-up was conducted in the 2014 wave. Frailty was assessed by a 40-item frailty index (FI) and classified into frail (FI > 0.21), prefrail (FI: 0.1–0.21), and non-frail (FI ≤0.1). Vitamin D was assessed by 25-Hydroxyvitamin D (25(OH)D) and categorized into quartiles and dichotomies (normal: ≥50 nmol/L vs. low: < 50 nmol/L). The presence of ≥20 natural teeth was defined as functional dentition, otherwise as non-functional dentition. We used bivariate logistic regression and restricted cubic splines to examine the association between vitamin D, dentition status, and frailty. We created a multiplicative interaction between vitamin D and dentition status to test for their combined effect.

**Results:**

A total of 205 (19.1%) incident frailty were identified during the 3-year follow-up. Participants with the lowest quartile of plasma 25(OH) D were more likely to be frail (odds ratio [OR] 2.45, 95% confidence interval [CI]: 1.38 to 4.35) than those in the highest quartile. Older adults with the lowest quartile of 25(OH) D and non-functional dentition had the highest odds of frailty (OR = 3.67, 95% CI: 1.02 to 13.12). We also observed that a lower vitamin D level was associated with an increased risk of frailty with a threshold of 40.37 nmol/L using restricted cubic spline models. However, vitamin D levels were not significantly associated with frailty among participants with functional dentition.

**Conclusions:**

Low vitamin D levels were associated with an increased risk of frailty in older adults. Functional dentition modified the association of vitamin D with frailty.

**Supplementary Information:**

The online version contains supplementary material available at 10.1186/s12877-022-02857-3.

## Background

Population aging is a major public health challenge globally [1]. One of the most significant problems of a rapidly aging population is frailty [2]. Frailty is an age-related geriatric syndrome characterized by reduced physiological reserve and high susceptibility to stress stimulation [2, 3]. Numerous studies have shown that frail older adults are predisposed to a broad range of adverse health outcomes, including falls [4], fractures [5], disability [6], cognitive decline [7], hospitalization [8], depression [9], loneliness [10], impaired quality of life [11], and death [12]. An increasing amount of studies suggest that identifying risk factors for frailty and implementing effective interventions can reverse frailty at an early stage [13].

Vitamin D maintains serum calcium and phosphate homeostasis to promote strong bones, regulates the innate immune system, cell differentiation, and cell maturation, and influences cellular metabolism of skeletal muscle [14, 15]. Older adults with 25(OH) D deficiency performed worse on functional capacity, such as timed walk, sit-to-stand test, and tandem stands than those with normal 25(OH) D levels [16]. Vitamin D deficiency was also associated with exhaustion and physical inactivity [17]. Longitudinal studies have shown that low vitamin D level is associated with increased risk of frailty [18, 19]. In addition, tooth loss is a common condition among older adults. Studies have reported that tooth loss and non-functional dentition (defined as <20 teeth) were associated with increased risk of frailty [20–22], while nutritional status appeared to mediate this association [23]. A prospective observational study revealed that lower serum 25(OH) D concentrations were associated with increased risk of periodontitis and tooth loss [24]. However, whether low vitamin D levels and dentition status have combined effects on frailty is still unclear. Furthermore, whether the association between vitamin D and frailty is modified by dentition status is unknown.

The purpose of the present study is to evaluate the combined effects of low vitamin D levels and dentition status on the risk of frailty among old adults. We also investigated whether the association between vitamin D and frailty is modified by dentition status.

## Methods

### Data sources and setting

The Chinese Longitudinal Healthy Longevity Survey (CLHLS) is an ongoing prospective, longitudinal study with the largest sample of the oldest old in China. It aims to identify the determinants of health and longevity among Chinese older adults. Half of the counties and municipalities were randomly selected in 23 of the 31 provinces, covering 85% of the national population. The baseline survey was conducted in 1998; alive participants were re-interviewed in follow-up surveys conducted every two or 3 years. The response rate was over 80%. Baseline data of the present study were obtained from a biomarker sub-study conducted in eight longevity regions in 2012: Laizhou City of Shandong Province, Xiayi County of Henan Province, Zhongxiang City of Hubei Province, Mayang County of Hunan Province, Yongfu County of Guangxi Autonomous Area, Sanshui District of Guangdong Province, Chengmai County of Hainan Province, and Rudong County of Jiangsu Province [25]. These eight regions accounted for one-third of China’s longevity areas, covering the southern, middle, and northern regions of the country [26]. More details of the CLHLS have been described in previous articles [27, 28].

### Study population

The inclusion criteria were the older people who were ≥ 65 years old and provided blood samples. The follow-up was conducted in the 2014 wave. Participants with any of the following conditions were excluded: (1) frailty at baseline; (2) missing data on key variables (25(OH) D level and/or natural tooth number); (3) lost to follow-up or death. The flow chart is shown in Supplementary Fig. S1, Additional file 1. Finally, we retained 1074 older participants who were non-frail or prefrail at baseline in the analytic sample.

### Outcomes

The outcome was the incident frailty during follow-up. Frailty status at baseline and follow-up was measured by the frailty index (FI)—a commonly used tool for assessing frailty. Following a standard procedure [29], we constructed the FI based on 40 items, including self-rated health, psychological characteristics, disability in activities of daily living, functional limitations, cognitive function, audition, vision, heart rhythm, chronic diseases, and interviewer-rated health status (Supplementary Table S1, Additional file 1). The binary variable was coded as 1 if a deficit was present and 0 otherwise. Ordinal variables were assigned scored from 0 to 1 to express health deficit. A higher value indicated a more severe health deficit. For example, health worsened in the past year was scored as follows: much better = 0; a little better = 0.25; no change = 0.5; a little worse = 0.75; much worse = 1. If the respondent had more than one serious illness or had been bedridden in the past 2 years, we assigned a score of 2. For each individual, the FI was calculated by dividing the observed number of deficits by the total possible number of deficits (41 deficits from 40 indicators), resulting in a score ranging from 0 to 1. If more than 15% of deficits were missing, the FI was considered missing. We defined the FI value > 0.21 as frailty, 0.10–0.21 as prefrailty, and ≤ 0.10 as nonfrailty [30].

### Risk factors

Vitamin D was assessed by plasma 25(OH)D. Trained medical personnels collected blood samples using heparin anticoagulant vacuum tubes from subjects who had fasted overnight. They centrifuged blood samples at 2500 rpm at 20 °C for 10 min, and measured plasma 25(OH) D levels by enzyme-linked immunosorbent assay (Immunodiagnostic Systems Limited, Bolton, UK). Plasma 25(OH) D was categorized into quartiles (< 31.78, 31.78–43.65, 43.65–56.61, and ≥ 56.61 nmol/L). Stratified analysis was analyzed at a dichotomous cut-off point of 25(OH) D deficiency recommended by the Endocrine Society Guidelines (Normal vitamin D level: ≥50 nmol/L vs. low vitamin D level: < 50 nmol/L) [31].

### Effect modifier

The number of remaining natural teeth was assessed at baseline by trained personnel based on a question: “How many natural teeth do you have?” Functional dentition was defined as the presence of ≥20 teeth which can assure an acceptable level of oral function [32], and we categorized natural teeth into dichotomies according to dentition status (functional dentition [≥20 natural teeth] and non-functional dentition [<20 natural teeth]).

### Covariates

Based on the literature [33–35], we chose the following covariates: age in years, sex (female vs. male), marital status (married vs. others [divorced, widowed, or never married]), education level (≥1 year of schooling vs. no schooling), residence (rural vs. urban), co-residence (with family member [s] vs. Others [living alone or in an institution]), economic independence (own labor income/having a retirement wage vs. others), currently smoking (yes vs. no), currently drinking (yes vs. no), regular exercise at present (yes vs. no), sleep time (≤5, 6 to 8, or ≥ 9 h), body mass index (BMI), calf circumference (< 31 cm vs. ≥31 cm), season of blood draw, high-sensitive C-reactive protein (hs-CRP), and plasma albumin levels. BMI (kg/m^2^) is calculated as weight in kilograms divided by height in meters squared. We classified BMI (kg/m^2^) into four categories (< 18.5, 18.5–24, 24–28, and ≥ 28 kg/m^2^) [36]. Plasma albumin and hs-CRP were measured in a continuous automatic analyzer (Hitachi 7108, Tokyo, Japan) using commercially available diagnostic kits (Roche Diagnostic). All blood biomarkers were tested by the central clinical laboratory of Capital Medical University.

### Statistical analysis

Continuous variables with skewed distribution were described using median (interquartile range, IQR). Categorical variables were expressed as frequency (percentage). Kruskal-Wallis test for continuous variables and chi-square test for categorical variables were applied to compare the differences in baseline characteristics.

Bivariate logistic regression analyses were used to assess the association of vitamin D and dentition status (functional dentition vs. non-functional dentition) with frailty. Multiplicative interaction was tested to evaluate effect modification by including a cross-product term for dentition status and plasma 25(OH) D in two different ways (dentition status [functional dentition and non-functional dentition] x vitamin D [quartile] and dentition status [functional dentition and non-functional dentition] x vitamin D [< 50 nmol/L and ≥ 50 nmol/L]) in the regression model. To observe the combined effect of vitamin D and dentition status on frailty, we created eight-level joint groups according to dentition status (functional dentition and non-functional dentition) and vitamin D quartiles. The subgroup analyses of the association of vitamin D with frailty were conducted by dentition status. Age, sex, baseline frailty status (non-frail and prefrail), residence, marital status, education level, economic independence, smoking status, drinking status, regular exercise, sleep time, BMI, calf circumference, and season of blood draw, albumin, and hs-CRP were included as covariates in adjusted models. The validity of the regression model was evaluated by calculating the tolerance and variance inflation factor (VIF) for multicollinearity diagnosis. Tolerance > 0.1 and VIF < 10 are used to indicate that there is no multicollinearity between the independent variables. Tests for trend across vitamin D quartiles were examined using ordinal values in separate models.

We used restricted cubic splines to evaluate the pattern and magnitude of associations between 25(OH) D levels and frailty risk in all participants and participants stratified by dentition status (functional dentition vs. non-functional dentition). The 75th percentile of 25(OH) D level was treated as the reference, and the 5th, 25th, 50th, 75th, and 95th percentiles of 25(OH) D were treated as the 5 knots for spline. The likelihood ratio test tested the potential nonlinearity.

The results were presented as odds ratios (ORs) with 95% confidence intervals (CIs). A two-tailed *P* value less than 0.05 was considered statistically significant. The analyses were performed using Stata 13.0 (Stata Corporation, College Station, TX).

## Results

### Participants’ general characteristics

Of 1074 participants included in the present study, 41.5% (*n* = 446) were prefrail and 58.5% (*n* = 628) were non-frail at baseline. The participants with higher plasma 25(OH) D levels were more likely to be younger, male, married, have a higher level of education and live in rural areas (Table 1). They were also more likely to be non-frail.

**Table 1:** Baseline characteristics by quartiles of vitamin D levels in the 2011–2012 wave

Characteristics	All participants	Plasma 25(OH) D concentrations				*P* value
		Quartile 1 (< 31.78 nmol/L)	Quartile 2 (31.78–43.65 nmol/L)	Quartile 3 (43.65–56.61 nmol/L)	Quartile 4 (≥56.61 nmol/L)	
Age, median (IQR), years	79 (71, 87)	81 (71, 91)	80 (72, 86)	79 (71, 86)	77 (70, 85)	0.012
Female, no. (%)	498 (46.4)	163 (60.8)	132 (49.1)	115 (42.9)	88 (32.7)	< 0.001
Married, no. (%)	583 (54.3)	121 (45.2)	146 (54.3)	153 (57.1)	163 (60.6)	0.003
Rural, no. (%)	880 (81.9)	221 (82.5)	202 (75.1)	221 (82.5)	236 (87.7)	0.002
Education (≥ 1 year), no. (%)	512 (47.7)	100 (37.3)	124 (46.1)	137 (51.1)	151 (56.1)	< 0.001
With household member(s), no. (%)	816 (76.0)	203 (75.8)	206 (76.6)	206 (76.9)	201 (74.7)	0.938
Economic independence, no. (%)	373 (34.7)	93 (34.7)	96 (35.7)	87 (32.5)	97 (36.1)	0.819
Smoking status						0.001
Current	843 (78.5)	230 (85.8)	217 (80.7)	201 (75.0)	195 (72.5)	
Never or former	231 (21.5)	38 (14.2)	52 (19.3)	67 (25.0)	74 (27.5)	
Drinking status						< 0.001
Current	866 (80.6)	229 (85.5)	229 (85.1)	213 (79.5)	195 (72.5)	
Never or former	208 (19.4)	39 (14.6)	40 (14.9)	55 (20.5)	74 (27.5)	
Regular exercise	207 (19.3)	42 (15.7)	50 (18.6)	56 (20.9)	59 (21.9)	0.262
Natural tooth number, no.						0.905
< 20	748 (69.7)	191 (71.3)	185 (68.8)	184 (68.7)	188 (69.9)	
≥20	326 (30.4)	77 (28.7)	84 (31.2)	84 (31.3)	81 (30.1)	
Total sleep time (h)						0.226
≤5	179 (16.7)	44 (16.4)	37 (13.8)	40 (14.9)	58 (21.6)	
6–8	708 (65.9)	178 (66.4)	179 (66.5)	182 (67.9)	169 (62.8)	
≥9	187 (17.4)	46 (17.2)	53 (19.7)	46 (17.2)	42 (15.6)	
BMI (kg/m^2^)						0.512
< 18.5	191 (17.8)	45 (16.8)	44 (16.4)	42 (15.7)	60 (22.3)	
18.5–24	615 (57.3)	156 (58.2)	153 (56.9)	160 (59.7)	146 (54.3)	
24–28	209 (19.5)	52 (19.4)	57 (21.2)	55 (20.5)	45 (16.7)	
≥28	59 (5.5)	15 (5.6)	15 (5.6)	11 (4.1)	18 (6.7)	
Calf circumference (< 31 cm)	524 (48.8)	144 (53.7)	144 (53.5)	127 (47.4)	109 (40.5)	0.006
Prefrail	446 (41.5)	139 (51.9)	113 (42.0)	96 (35.8)	98 (36.4)	< 0.001
Season of blood draw						< 0.001
Spring (March–May)	263 (24.5)	108 (40.3)	65 (24.2)	48 (17.9)	42 (15.6)	
Summer (June–August)	745 (69.4)	158 (59.0)	201 (74.7)	204 (76.1)	182 (67.7)	
Autumn (September–November)	66 (6.2)	2 (0.8)	3 (1.1)	16 (6.0)	45 (16.7)	
25(OH) D (nmol/L)	43.65 (31.78, 56.61)	25.83 (21.26, 28.84)	37.29 (34.10, 40.73)	49.70 (46.65, 52.80)	67.21 (61.15, 75.95)	< 0.001
Albumin (g/L)	41.36 (38.40, 44.30)	40.50 (37.20, 43.48)	41.90 (39.10, 44.60)	41.50 (38.30, 44.88)	41.40 (38.90, 44.00)	0.002
hs-CRP (mg/L)	0.79 (0.37, 1.90)	0.86 (0.38, 2.08)	0.72 (0.34, 1.72)	0.80 (0.38, 1.83)	0.81 (0.38, 1.99)	0.444

*BMI* Body mass index, *25(OH) D* 25-Hydroxyvitamin D, *hs-CRP* high-sensitive C-reactive protein

Notes: *P* values from Kruskal–Wallis test for continuous variables and chi-square test for categorical variables

### Association between vitamin D levels and frailty

A total of 205 (19.1%) incident frailty were identified during the 3-year follow-up. Participants with low vitamin D levels had a significantly higher frailty risk than those with normal vitamin D levels (22.7% and 12.8%, respectively; *P* = 0.001). Table 2 presents the association between vitamin D levels and frailty. The odds of frailty in the lowest quartile of plasma 25(OH) D was 2.45 times the odds in the highest quartile after adjusting for confounding variables in Model 3.

**Table 2:** The association between plasma vitamin D concentrations and frailty

	Unadjusted Model		Model 1		Model 2		Model 3	
	OR (95% CI)	*P* value	OR (95% CI)	*P* value	OR (95% CI)	*P* value	OR (95% CI)	*P* value
Quartiles of 25(OH)D^a^								
Quartile 1 (< 31.78 nmol/L)	3.04 (1.94, 4.77)	< 0.001	2.56 (1.53, 4.28)	< 0.001	2.46 (1.39, 4.36)	0.002	2.45 (1.38, 4.35)	0.002
Quartile 2 (31.78–43.65 nmol/L)	1.63 (1.01, 2.63)	0.044	1.81 (1.06, 3.09)	0.030	1.69 (0.94, 3.01)	0.079	1.68 (0.94, 3.00)	0.082
Quartile 3 (43.65–56.61 nmol/L)	1.33 (0.81, 2.17)	0.256	1.47 (0.85, 2.55)	0.172	1.33 (0.75, 2.37)	0.337	1.32 (0.74, 2.36)	0.342
Quartile 4 (≥56.61 nmol/L)	Reference		Reference		Reference		Reference	
*P* for trend	< 0.001		0.003		0.013		0.014	

*25(OH) D* 25-Hydroxyvitamin D, *OR* Odds ratio, *CI* Confidence interval

Notes: Model 1: adjusted for age, sex, and baseline frailty status (prefrailty vs. nonfrailty);

Model 2: further adjusted for residence, marital status, education level, economic independence, smoking status, drinking status, regular exercise, sleep time, body mass index, dentition status, calf circumference, and season of blood draw;

Model 3: further adjusted for albumin and high-sensitivity C-reactive protein

^a^Sample sizes: quartiles 1–4: 268, 269, 268, and 269, respectively

**Fig. 1:**
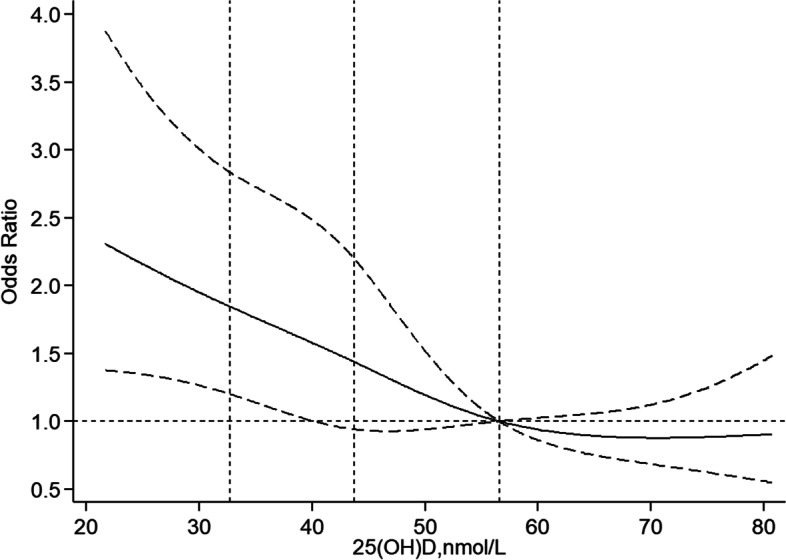
Association between vitamin D levels and frailty. The estimated odds ratios are indicated by the solid line and the 95% confidence intervals by dashed lines. The vertical dashed lines represent the first, second, and third quartiles of 25(OH)D. Reference is the 75th percentile of 25(OH)D. The model was adjusted for age, sex, baseline frailty status (prefrailty vs. nonfrailty), residence, marital status, education level, economic independence, smoking status, drinking status, regular exercise, sleep time, body mass index, dentition status, calf circumference, season of blood draw, albumin, and high-sensitivity C-reactive protein

**Fig. 2:**
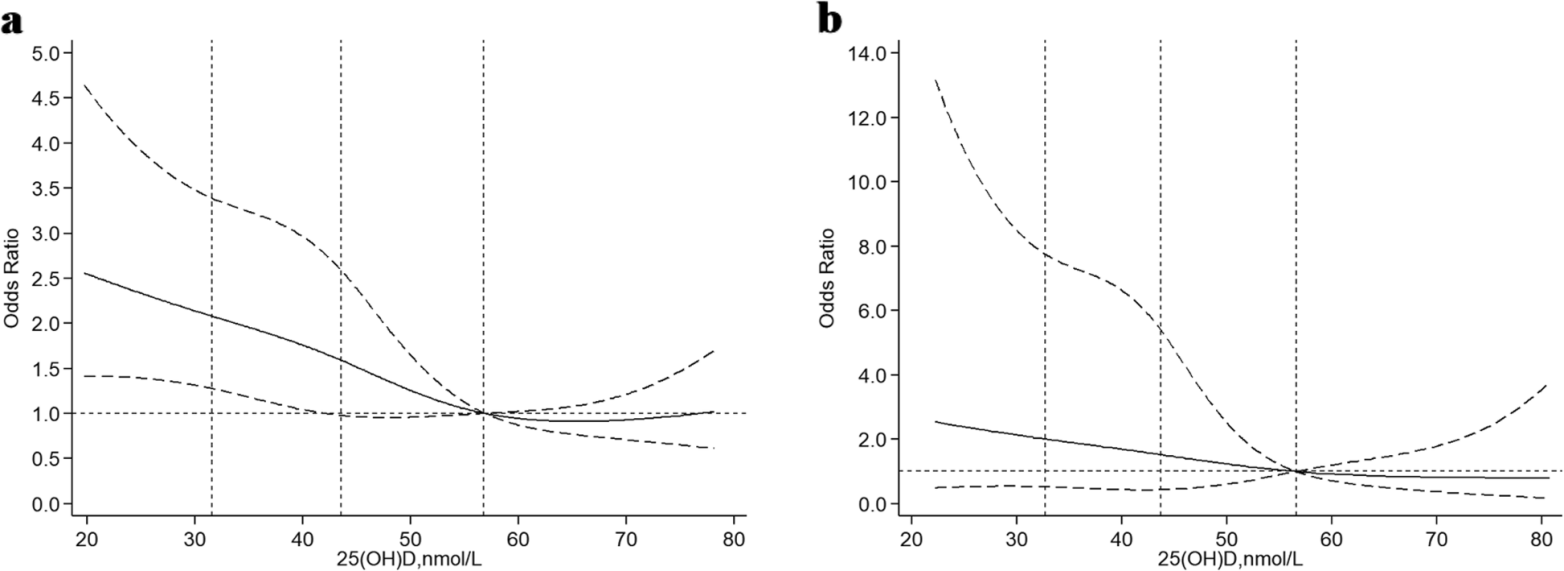
Association between vitamin D levels and frailty according to dentition status. (**a**) non-functional dentition; (**b**) functional dentition. The estimated odds ratios are indicated by the solid line and the 95% confidence intervals by dashed lines. The vertical dashed lines represent the first, second, and third quartiles of 25(OH)D. Reference is the 75th percentile of 25(OH)D. The model was adjusted for age, sex, baseline frailty status (prefrailty vs. nonfrailty), residence, marital status, education level, economic independence, smoking status, drinking status, regular exercise, sleep time, body mass index, dentition status, calf circumference, season of blood draw, albumin, and high-sensitivity C-reactive protein

Figure 1 shows the smoothed spline curve of ORs for vitamin D as continuous variables. The fully adjusted smooth curve indicated that the association of vitamin D with frailty was linear (P for nonlinearity = 0.344). There was a steep increase in the odds of frailty with decreasing vitamin D for concentrations < 40.37 nmol/L. The shape of the curve for the association between plasma vitamin D levels and frailty was significantly modified by dentition status (Fig. 2). For participants with non-functional dentition, vitamin D concentrations < 43.27 nmol/L were significantly inversely associated with frailty (Fig. 2a). However, vitamin D levels were not significantly associated with frailty for participants with functional dentition (Fig. 2b).

### The combined effect of vitamin D levels and dentition status on frailty

**Fig. 3:**
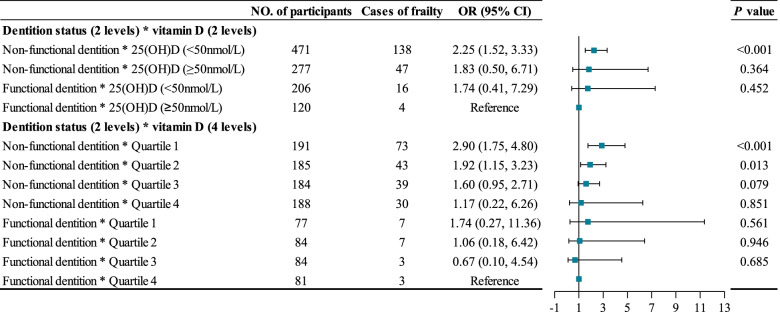
Interaction of dentition status with vitamin D levels on frailty. *25(OH) D* 25-Hydroxyvitamin D, *OR* odds ratio, *CI* confidence interval. Notes: Adjusted for age, sex, baseline frailty status (prefrailty vs. nonfrailty), residence, marital status, education level, economic independence, smoking status, drinking status, regular exercise, sleep time, body mass index, calf circumference, the season of blood draw, albumin, and high-sensitivity C-reactive protein. Quartile 1: 25-Hydroxyvitamin D < 31.78 nmol/L; Quartile 2: 31.78 nmol/L ≤ 25-Hydroxyvitamin D < 43.65 nmol/L; Quartile 3: 43.65 nmol/L ≤ 25-Hydroxyvitamin D < 56.61 nmol/L; Quartile 4: 25-Hydroxyvitamin D ≥ 56.61 nmol/L

Participants with the lowest quartile of 25(OH) D and non-functional dentition were 3.67 times more likely to develop frailty than those with the highest quartile of 25(OH) D and functional dentition (Table 3). In the subgroup, we noted a significant association between vitamin D and frailty among participants with non-functional dentition after adjusting for the confounding variables in Model 3 (Table 4). However, this association failed to reach statistical significance among those with functional dentition. A significant interaction between vitamin D and dentition status was observed in terms of frailty risk (P for interaction < 0.001) (Fig. 3).

**Table 3:** Odds ratios for the combined associations of vitamin D and dentition status with frailty

Vitamin D and dentition status	Unadjusted Model		Model 1		Model 2		Model 3	
	OR (95% CI)	*P* value	OR (95% CI)	*P* value	OR (95% CI)	*P* value	OR (95% CI)	*P* value
Quartile 1 + non-functional dentition (*n* = 191)	16.09 (4.90, 52.85)	< 0.001	4.13 (1.19, 14.40)	0.026	3.72 (1.04, 13.37)	0.044	3.67 (1.02, 13.12)	0.046
Quartile 2 + non-functional dentition (*n* = 185)	7.87 (2.37, 26.21)	0.001	2.82 (0.81, 9.85)	0.105	2.47 (0.69, 8.82)	0.164	2.42 (0.68, 8.65	0.174
Quartile 3 + non-functional dentition (*n* = 184)	6.99 (2.09, 23.36)	0.020	2.45 (0.69, 8.62)	0.164	2.05 (0.57, 7.37)	0.269	2.02 (0.56, 7.26)	0.280
Quartile 4 + non-functional dentition (*n* = 188)	4.94 (1.46, 16.68)	0.010	1.50 (0.42, 5.37)	0.537	1.42 (0.39, 5.23)	0.597	1.40 (0.38, 5.16)	0.611
Quartile 1 + functional dentition (*n* = 77)	2.60 (0.65, 10.44)	0.178	1.83 (0.43, 7.78)	0.411	1.51 (0.34, 6.67)	0.584	1.49 (0.34, 6.57)	0.599
Quartile 2 + functional dentition (*n* = 84)	2.36 (0.59, 9.48)	0.225	1.57 (0.37, 6.61)	0.538	1.25 (0.29, 5.37)	0.766	1.25 (0.29, 5.40)	0.762
Quartile 3 + functional dentition (*n* = 84)	0.96 (0.19, 4.92)	0.964	0.74 (0.14, 3.95)	0.728	0.68 (0.13, 3.67)	0.653	0.68 (0.13, 3.65)	0.649
Quartile 4 + functional dentition (*n* = 81)	Reference		Reference				Reference	

*OR* Odds ratio, *CI* Confidence interval

Model 1: adjusted for age, sex, and baseline frailty status (prefrailty vs. nonfrailty);

Model 2: further adjusted for residence, marital status, education level, economic independence, smoking status, drinking status, regular exercise, sleep time, body mass index, calf circumference, and the season of blood draw;

Model 3: further adjusted for albumin and high-sensitivity C-reactive protein;

Quartile 1: 25-Hydroxyvitamin D < 31.78 nmol/L; Quartile 2: 31.78 nmol/L ≤ 25-Hydroxyvitamin D < 43.65 nmol/L; Quartile 3: 43.65 nmol/L ≤ 25-Hydroxyvitamin D < 56.61 nmol/L; Quartile 4: 25-Hydroxyvitamin D ≥ 56.61 nmol/L

**Table 4:** Odds ratios for the association between vitamin D and frailty by dentition status

Group	Unadjusted Model		Model 1		Model 2		Model 3	
	OR (95% CI)	*P* value	OR (95% CI)	*P* value	OR (95% CI)	*P* value	OR (95% CI)	*P* value
Functional dentition								
Low vitamin D ^a^ (*n* = 206)	2.44 (0.80, 7.48)	0.118	1.68 (0.52, 5.49)	0.388	1.64 (0.40, 6.72)	0.492	1.74 (0.41, 7.29)	0.452
Normal vitamin D ^b^ (*n* = 120)	Reference		Reference		Reference		Reference	
Non-functional dentition								
Low vitamin D ^a^ (*n* = 471)	2.03 (1.40, 2.94)	< 0.001	2.07 (1.37, 3.15)	0.001	2.13 (1.34, 3.39)	0.001	2.13 (1.33, 3.39)	0.002
Normal vitamin D ^b^ (*n* = 277)	Reference		Reference		Reference		Reference	

*OR* Odds ratio, *CI* Confidence interval

Model 1: adjusted for age, sex, and baseline frailty status (prefrailty vs. nonfrailty);

Model 2: further adjusted for residence, marital status, education level, economic independence, smoking status, drinking status, regular exercise, sleep time, body mass index, dentition status, calf circumference, and season of blood draw;

Model 3: further adjusted for albumin and high-sensitivity C-reactive protein;

^a^25-Hydroxyvitamin D < 50 nmol/L; ^b^ 25-Hydroxyvitamin D ≥ 50 nmol/L

## Discussion

In this nationwide cohort study of 1, 074 Chinese older adults, we observed that the odds of frailty in the lowest quartile of plasma 25(OH) D was higher than that in the highest quartile, and participants with the lowest quartile of 25(OH) D and non-functional dentition had significantly higher odds of frailty. In addition, we found functional dentition modified the association of vitamin D with frailty. Specifically, among participants with non-functional dentition, low vitamin D concentrations were associated with increased frailty risk, whereas there were no significant relationships among those with functional dentition.

Our study was in agreement with previous studies that showed an inverse correlation between vitamin D and frailty [18, 37, 38]. In a large (*n* = 4, 203) population-based study of older men aged 70–88 years, the risk of frailty increased by 50% during 5 year-follow up for participants who were not frailty at baseline and had low levels of vitamin D (< 52.9 nmol/L) [18]. In another prospective analysis based on the Germany cohort with a 2.9-year follow-up, deficient levels of 25(OH)D(< 15 ng/ml) were independently associated with incident pre-frailty as well as incident frailty [37]. Vitamin D exerts some molecular effects within muscle cell and has anti-inflammatory properties, which might lead to lower risk of frailty. Specifically, vitamin D regulates calcium flux, mineral homeostasis, and protein anabolic signaling pathways in muscle tissue [39], which may be beneficial for maintaining muscle mass, strength, and contraction. Our findings showed that the further adjustment of hs-CRP in model 3 did not attenuate the association between plasma 25(OH) D concentration and frailty. The possible explanation is that frailty is a process with persistent chronic inflammation, whereas hs-CRP is a marker of acute systemic inflammation. Therefore, the immunologic and inflammatory mechanisms involved in the course of acute systemic inflammation are different from those involved in frailty. Vitamin D regulates inflammation in muscles and may not be enough to alter circulating inflammatory marker levels. Even so, it has been well established that active vitamin D metabolites can downregulate intramuscular inflammation [40, 41]. Vitamin D3 supplementation may directly suppress exercise-induced intramuscular inflammation [40]. Increased circulating 25(OH) D concentrations contribute to muscle recovery from injury by suppressing intramuscular inflammation [42]. However, none of these previous studies considered the modification effect of dentition status on the association between vitamin D levels and frailty.

Natural tooth number is a risk indicator of frailty as well as vitamin D [43, 44]. A prospective study found that one more tooth was associated with 5.0% lower odds of frailty in 237 older Mexican adults during 3 year-follow up [43]. A longitudinal cohort study of older British men with a 3-year follow-up demonstrated that edentulous was associated with high risks of developing frailty (OR = 1.90, 95% CI: 1.03 to 3.52) [44]. We extended previous research by examining the role of dentition status in the association between vitamin D and frailty. Our study found that participants with low plasma 25(OH) D concentrations and non-functional dentition were at a much greater risk of frailty. In addition, this joint effect was significant on the multiplicative level. Interestingly, the shape of the curve for the association between plasma vitamin D levels and frailty was significantly modified by dentition status, and vitamin D levels were not significantly associated with frailty among participants with functional dentition. Our findings suggest that dentition status may be a good marker in identifying subgroups of older adults with low vitamin D levels that are potentially more harmful. However, the causal pathway remains unclear. In this study, albumin concentrations were significantly higher in participants with functional dentition than in those with non-functional dentition (42.1 ± 4.6 g/L vs. 40.9 ± 4.4 g/L). One possible mechanism is that tooth loss leads to difficulty in chewing and fatigue, which might result in reduced consumption of fruits, vegetables, protein, and micronutrients, and increased carbohydrate intake. These changes in dietary patterns could lead to an increased risk of malnutrition that is strongly associated with frailty [45–47]. An additional consideration is that the lower number of teeth may be associated with an adaptation of the diet of older people. This adaptation may represent an inadequate consumption of vitamin-D rich food. However, we did not find a significant association between vitamin D and dentition status. It is also plausible that tooth loss has adverse effects on social interaction and self-esteem [48, 49], leading to frailty [50, 51]. Furthermore, the inverse association between plasma 25(OH) D concentration and frailty was only found in participants who had no functional dentition, is it highly possible that low plasma 25(OH) D concentration is a biomarker of deficits of other nutrients as a result of impaired functional dentition. Meanwhile, the potential influence of residual confounding could not be excluded. Taken together, functional dentition may help reduce the risk of frailty. Our results provide evidence to support simultaneous screening for vitamin D levels and dentition status in older adults.

Frailty is likely to be even more acute in China, which has the largest aging population in the world. In China, studies have shown that the prevalence of pre-frailty and frailty among people aged 60 years and above is 51.2 and 7.0%, respectively [52]. Frailty may be modifiable and considered more reversible than disability but may not be recoverable when it becomes a pre-death stage. Therefore, strategies to prevent and slow down frailty development are critical. Low 25(OH) D levels and tooth loss are strongly associated with frailty, so maintaining adequate levels of 25(OH) D and maintaining functional dentition throughout life can improve health conditions such as frailty with age.

### Strengthen and limitation

To our knowledge, this was the first study to examine an interactive effect of vitamin D levels and dentition status on frailty. Our longitudinal study used the frailty index to assess the relationship between vitamin D, dentition status, and frailty as it evaluates a broader range of diseases (such as cognitive function) than a frailty phenotype and may provide additional information to explore correlations. Nevertheless, we recognize several limitations of this study. First, our participants were Chinese older than 65 years of age. Our results have limited generalizability to other ethnic groups. Second, dentition status and 25(OH) D levels were assessed at baseline only. Thus, tooth loss and 25(OH) D concentration changes during the observation period may confuse the results. In future studies, it will be essential to examine the effect of these changes. Third, because CLHLS is not explicitly designed for dental research, other information related to oral health is lacking. The number of teeth was self-reported, and the reliability of the numbers was limited. When exploring the deeper relationship between oral health and frailty, dentition status may not be an adequate proxy for oral function. Finally, as with any analysis, residual confusion due to non-measured factors cannot be ruled out.

## Conclusions

We found that low vitamin D levels are associated with an increased risk of frailty. We also found that functional dentition modified the association between vitamin D levels and frailty. These results suggest that 25(OH) D levels and dentition status can be early warning indicators of frailty. It would lead to more aggressive interventions to prevent and treat frailty in older people who have a combination of significant tooth loss and vitamin D deficiency. Additional research is needed to investigate how to maintain functional dentition through oral care, combined with nutritional interventions aimed at optimizing plasma 25(OH) D concentrations, to potentially reduce the occurrence of frailty.

## Supplementary Information

**Additional file 1.**

## Data Availability

The data that support the findings of this study are available from Peking University but restrictions apply to the availability of these data, which were used under license for the current study, and so are not publicly available. Data are however available from the authors upon reasonable request and with permission of Peking University.

## Abbreviations

25(OH)D: 25-Hydroxyvitamin D
OR: Odds ratio
CI: Confidence interval
CLHLS: Chinese Longitudinal Healthy Longevity Survey
FI: Frailty index
BMI: Body mass index
hs-CRP: High-sensitive C-reactive protein
IQR: Interquartile range
VIF: Variance inflation factor
