# Supplementary material for: Functional dentition is a modifier of the association between vitamin D and the frailty index among Chinese older adults: a population-based longitudinal study
Source: BMC Geriatr. 2022 Feb 28;22:159. doi: 10.1186/s12877-022-02857-3 (PMC8883641; doi:10.1186/s12877-022-02857-3)
Supplement: Supplementary file 1 — Additional file 1. [file 12877_2022_2857_MOESM1_ESM.docx]

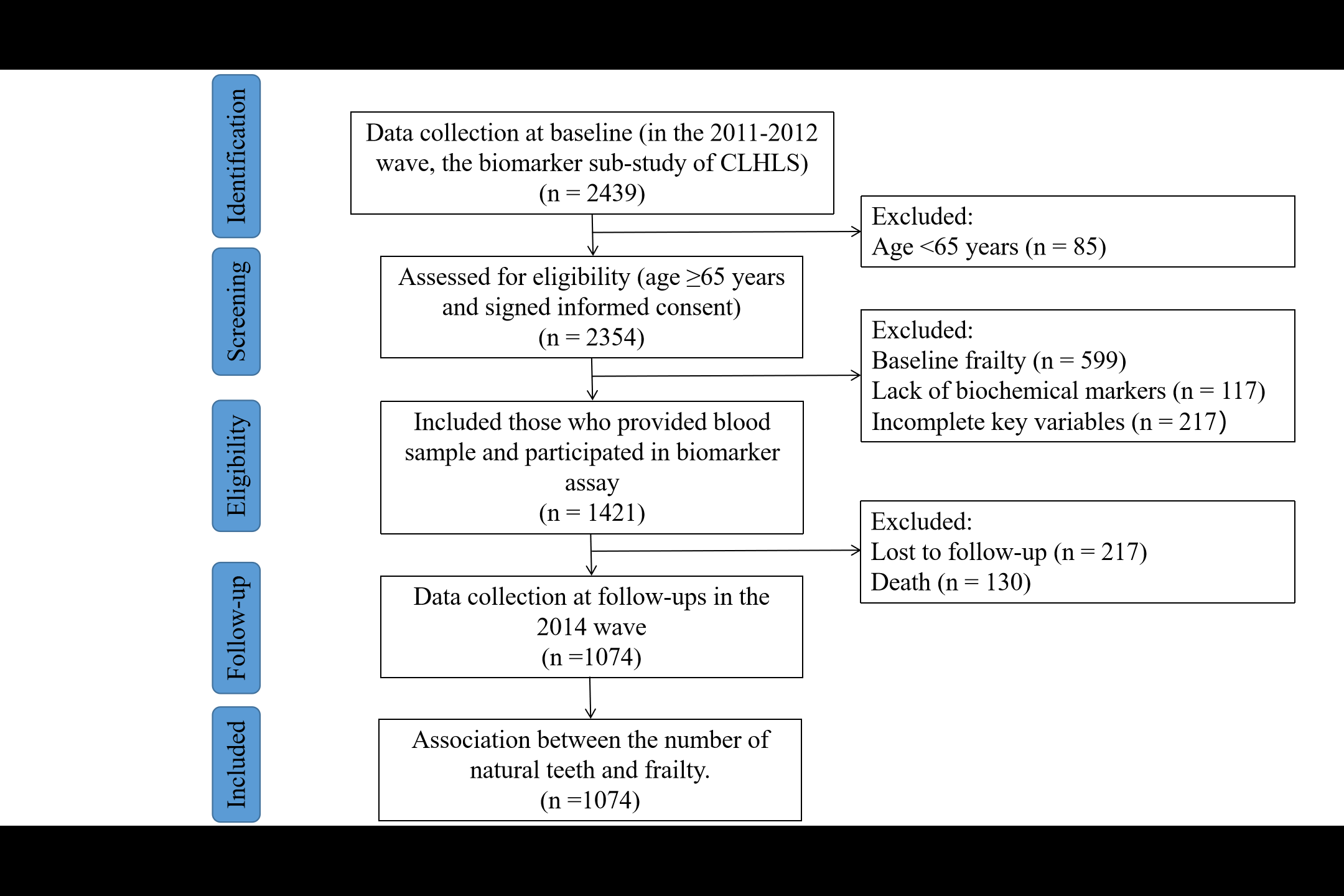


**Figure S1** Flowchart of the included study population.

*CLHLS* Chinese Longitudinal Healthy Longevity Survey.

**Table S1** Health variables and cut-points for the frailty index

| **NO.** | **Items** | **Cut point** |
| --- | --- | --- |
| 1 | Self-rated health | Very bad = 1, Bad= 0.75, So so = 0.5, Good = 0.25, Very good = 0 |
| 2 | Health worsened in the past year | Much worse = 1, A little worse = 0.75, No change = 0.5, A little better = 0.25, Much better = 0 |
| 3 | Feel fearful or anxious | Always = 1, Often =0.75, Sometimes = 0.5, Seldom = 0.25, Never = 0 |
| 4 | Feel lonely and isolated | Always = 1, Often =0.75, Sometimes = 0.5, Seldom = 0.25, Never = 0 |
| 5 | Feel useless with age | Always = 1, Often =0.75, Sometimes = 0.5, Seldom = 0.25, Never = 0 |
| 6 | Cognitively impaired (based on the Mini Mental State Examination) | ≤10 = 1, 11-17 = 0.75, 18-20 = 0.5, 21-24 = 0.25, ≥25 = 0 |
| 7 | ADLs: Needs assistance bathing | More than one part assistance = 1, Partial assistance = 0.5, Without assistance = 0 |
| 8 | ADLs: Needs assistance dressing | Assistance in getting clothes and getting dressed = 1, Need assistance for trying shoes = 0.5, Without assistance = 0 |
| 9 | ADLs: Needs assistance toileting | Don't use toilet = 1, Assistance in cleaning or arranging clothes = 0.5, Without assistance = 0 |
| 10 | ADLs: Needs assistance in indoor transferring | Bedridden = 1 , With assistance = 0.5, Without assistance = 0 |
| 11 | ADLs: Incontinence | Incontinent = 1, Occasional accidents = 0.5, Without assistance = 0 |
| 12 | ADLs: Needs assistance eating | Need feeding = 1, With some help = 0.5, Without assistance = 0 |
| 13 | IADLs: able to visit neighbors by oneself | Unable to do so = 1, A little difficult = 0.5, Yes = 0 |
| 14 | IADLs: able to shop by oneself if necessary | Unable to do so = 1, A little difficult = 0.5, Yes = 0 |
| 15 | IADLs: able to cook meals by oneself if necessary | Unable to do so = 1, A little difficult = 0.5, Yes = 0 |
| 16 | IADLs: able to wash clothing by oneself | Unable to do so = 1, A little difficult = 0.5, Yes = 0 |
| 17 | IADLs: able to walk continuously for 1 kilometer | Unable to do so = 1, A little difficult = 0.5, Yes = 0 |
| 18 | IADLs: able to lift a weight of 5 kg (such as a heavy bag of groceries) | Unable to do so = 1, A little difficult = 0.5, Yes = 0 |
| 19 | IADLs: able to continuously crouch and stand up three times | Unable to do so = 1, A little difficult = 0.5, Yes = 0 |
| 20 | IADLs: able to use public transportation | Unable to do so = 1, A little difficult = 0.5, Yes = 0 |
| 21 | Able to use chopsticks to eat | No = 1, Yes = 0 |
| 22 | Able to put hand behind neck | Neither hand = 1, Right or left hand only = 0.5, Both hands = 0 |
| 23 | Able to put hand behind lower back | Neither hand = 1, Right or left hand only = 0.5, Both hands = 0 |
| 24 | Able to raise arm upright | Neither hand = 1, Right or left hand only = 0.5, Both hands = 0 |
| 25 | Able to stand up from sitting in a chair | No = 1, Yes, using hands = 0.5, Yes, without using hands = 0 |
| 26 | Able to pick up a book from the floor | No = 1, Yes, sitting = 0.5, Yes, standing = 0 |
| 27 | Abnormal heart rhythm | Yes = 1, No = 0 |
| 28 | Vision loss | Can't see or blind = 1, Can see only = 0.5, Can see and distinguish = 0 |
| 29 | Hearing loss | Yes = 1, No = 0 |
| 30 | Number of serious illnesses in the past 2 years | Two or more illnesses or bedridden = 2, one illness = 1, No = 0 |
| 31 | Suffering from hypertension | Yes = 1, No = 0 |
| 32 | Suffering from diabetes | Yes = 1, No = 0 |
| 33 | Suffering from heart disease | Yes = 1, No = 0 |
| 34 | Suffering from stroke/cerebrovascular disease | Yes = 1, No = 0 |
| 35 | Suffering from bronchitis, emphysema, asthma, or pneumonia | Yes = 1, No = 0 |
| 36 | Suffering from tuberculosis | Yes = 1, No = 0 |
| 37 | Suffering from cancer | Yes = 1, No = 0 |
| 38 | Suffering from Parkinson's disease | Yes = 1, No = 0 |
| 39 | Suffering from arthritis | Yes = 1, No = 0 |
| 40 | Poor interviewer-rated health | Yes = 1, No = 0 |

*ADLs* activities of daily, *IADLs* instrumental activities of daily living.
